# Beyond the Machine: An Integrative Framework of Anthropomorphism in AI

**DOI:** 10.3390/bs16030358

**Published:** 2026-03-03

**Authors:** Petru Lucian Curșeu, Ștefana Radu

**Affiliations:** 1 Department of Psychology, Babeș-Bolyai University, 400015 Cluj-Napoca, Romania; stefana.radu@stud.ubbcluj.ro; 2 Department of Organization, Open Universiteit, 6419 AT Heerlen, The Netherlands

**Keywords:** artificial intelligence, anthropomorphism, theory of planned behavior, technology acceptance model, threat rigidity model, AI threats, AI opportunities

## Abstract

AI-enabled technology (AI) has a transformational role in our modern society because it is increasingly used as an interaction partner, making anthropomorphism (tendency to ascribe human features to non-human agents) a central mechanism shaping how people evaluate, accept or resist AI systems. Existing technology acceptance models and anthropomorphism frameworks, however, offer limited guidance on how human-like attributes of AI translate into perceptions of usefulness, perceived control, perceived opportunity or threats, particularly across different levels of AI autonomy. Building on the theory of planned behavior, the technology acceptance model and threat rigidity model, this paper develops a mid-range conceptual framework of AI anthropomorphism grounded in universal social perception dimensions of warmth and competence. We integrate fragmented research to derive three core propositions and four corollaries that specify how warmth and competence attributions shape evaluative cognitions in relation to AI. The framework further identifies AI autonomy as a boundary condition under which anthropomorphic cues may either facilitate acceptance or trigger perceptions of pseudo-empathy, cognitive superiority and identity threat. By offering a parsimonious, theoretically informed model, this paper clarifies when anthropomorphism fosters acceptance versus resistance in human–AI interaction and provides a structured agenda for future empirical research and AI design aimed at fostering synergies and resilience in human–AI ecosystems.

## 1. Introduction

Artificial intelligence (AI) has become an integral part of modern life, serving both utilitarian and hedonic purposes. Voice assistants such as Apple’s Siri and Amazon’s Alexa, for example, not only facilitate efficient data processing and provide friendly recommendations, but also function as attractive brand interfaces (McLean et al., 2021) and entertaining companions for customers (Shao & Kwon, 2021). Smart driving or diagnostic systems have also brought consistent innovation directed toward simplifying human life, so much as saving it (Secinaro et al., 2021), organizations use hybrid human-AI teams to enhance performance (Harris-Watson et al., 2023), while modern cities are infused with technology aimed at improving the effectiveness of their social and economic governance (Curșeu et al., 2021). In turn, hedonism is reflected in the use of AI for the sole purpose of relaxation and amusement, as in the case of chatbots and artificially generated products such as the Metaverse. This “surplus reality” (Moreno, 1965) might allow future generations to escape the mundane and live solely through the lenses of AI. Fundamentally, AI is an interaction partner, and as such, it is often ascribed human attributes.

Computers as social actors theory (CSAT, Nass et al., 1995) states that in human–computer interactions, participants tend to automatically ascribe attributes to technology as they do in interpersonal interactions; in other words, AI is likely to be treated as a real human interlocutor. Especially because AI has infiltrated many domains of human life, we tend to treat AI as a real social actor sharing human traits and humane behaviors, a process known as anthropomorphism (Belloni & Curșeu, 2025; J. Kim & Im, 2023). Anthropomorphism is conceptualized from three different perspectives: “a technological stimulus, a tendency, and a perception” (M. Li & Suh, 2022, p. 2). The first definition touches on the potential of machines to exhibit a function close to human’s, through machine learning and natural language processing (Troshani et al., 2021), while the second one refers to a natural tendency in machine–user interactions to anthropomorphize the AI (M. Li & Suh, 2022). Lastly, the perspective most suitable for the present paper sheds light on the degree to which an AI system is perceived, both mindfully and mindlessly (Araujo, 2018), as having agentic, moral and human-like characteristics (Belloni & Curșeu, 2025; Olar et al., 2026).

Although anthropomorphic tendencies emerge at the interface of interacting with a broad range of technologies and systems, AI differs in that it is attributed with cognitive agency, autonomy and intentionality, key features that make it the ultimate tool capable of replacing the very cognitive functions that created it. AI therefore differs from other systems because it is perceived not just as a tool but as an autonomous, adaptive actor and, in some instances, even sociopathic (Belloni & Curșeu, 2025; Olar et al., 2026). Systematic and integrative reviews on AI anthropomorphism document substantial heterogeneity in how anthropomorphism is defined, operationalized and linked to specific outcomes such as technology acceptance, intention to use or trustworthiness (Chaturvedi et al., 2025; M. Li & Suh, 2022; T. Li et al., 2025; Ramaul et al., 2026). A similar conclusion across these reviews is that AI anthropomorphism can produce both beneficial and adverse consequences, yet the field lacks clear theoretical frameworks specifying when anthropomorphic cues facilitate acceptance and when they backfire (Chaturvedi et al., 2025). We build on CSAT (Nass et al., 1995), to argue that its anthropomorphic features shape human behavioral tendencies in relation to AI (J. Kim & Im, 2023). Building on key insights of the theory of planned behavior (TPB, Ajzen, 1991), technology acceptance model (TAM, Davis, 1989; Davis et al., 1989) and threat rigidity model (TRM, Staw et al., 1981), we then summarize the key dimensions of technology acceptance and intention to use. If AI is treated as a social partner, it means that social perception tendencies and interaction expectations are paramount to understand human–AI interaction.

In this conceptual paper, we build on the most universal trends in interpersonal perception and social cognition, namely the stereotype content model (SCM, Cuddy et al., 2008; Fiske et al., 2007), to derive an integrated framework for clustering the anthropomorphic attributes ascribed to AI. As interpersonal attitudes are key predictors of behavioral intentions, we intend to identify and resolve some inconsistencies in the literature by introducing a series of theoretical propositions related to the anthropomorphic characteristics of AI in relation to behavioral intentions. In doing so, we aim to make two key contributions. First, we aim to integrate the disparate literature on AI anthropomorphism in a comprehensive framework and formulate research propositions that can guide future research and assessment in the field. Second, we aim to provide actionable insights for professionals working with AI, in order to improve interaction quality and acceptability of AI in various work domains and situations.

### 1.1. Why Is AI Anthropomorphism Important?

Anthropomorphism has been described as a general human tendency (Epley et al., 2007) as people tend to ascribe human-like features to a variety of non-human agents. Because the feeling of social presence (a sensation of personal, sociable, and empathetic human interaction) is highly valued and searched for in our species, we argue that interactive artificial agents such as AI-powered systems are susceptible to anthropomorphism due to the salience of human-like interactions (McKee et al., 2023). Unlike static or purely instrumental technologies and systems, AI is often experienced as a responsive and adaptive social partner, capable of learning, reasoning and making decisions, without direct human intervention or control. Capacity for autonomous action means that anthropomorphic cues in AI (e.g., voice, language style, facial features) do not merely make the system appear more user-friendly, but instead activate AI-specific inferences about trustworthiness and moral agency, features that trigger perceptions of both opportunities and threats in human–AI interaction. In this sense, AI anthropomorphism is not merely an extension of human tendencies toward anthropomorphizing inanimate objects and systems; it is distinctive, as it shapes relational expectations of collaboration, delegation and potentially competing with AI. Lee et al. (2015) assert that in the domain of AI, users may unconsciously disregard the artificial nature of a system if they find sufficient reasons to perceive the interaction as one between humans. In a recent study that discussed the human-like attributes ascribed to AI, J. Kim and Im (2023) show that appearance, cognitive intelligence and emotional intelligence are the key dimensions used to anthropomorphize AI.

At the individual level, the role of anthropomorphism as an antecedent of social presence underscores our inclination to perceive genuine agency even in instances that virtually lack human attributes (Epley et al., 2007). Depending on the nature of human–AI interaction, we need to distinguish between *assistive AI systems* (e.g., chatbots, recommendation tools), which support but do not replace human decisions, and *autonomous AI systems* (e.g., self-driving cars, algorithmic hiring), which make independent decisions. This distinction is central because anthropomorphic cues may foster trust and ease of use in assistive contexts, but trigger suspicion or identity threat in autonomous contexts.

On a broader scale, with various industries developing and implementing personal AI assistants, it becomes imperative to develop methods for fostering enhanced emotional engagement and acceptance of AI. Such efforts aim to augment the likelihood of facilitating assistance and encouraging adherence to virtual assistants’ recommendations (Adam et al., 2021). In their integrative review on anthropomorphic tendencies, Epley et al. (2007) state that effectance (need for controlling the environment and effective interaction) and sociality (need for social connectivity) are two key motivational mechanisms that trigger humans to ascribe human-like attributes when various forms of agent-specific information are present (social cues, physical features, voice, etc.). Through anthropomorphism, human–AI interactions fulfil sociality needs through meaningful and authentic communication as well as effectance needs through understanding the AI interlocutor and jointly controlling the environment (solving problems, making decisions, etc.) (Epley et al., 2008). In relation to the use of technology, we argue that three theoretical frameworks, namely TPB (Ajzen, 1991), TAM (Davis, 1989) and TRM (Staw et al., 1981) (henceforth globally referred to as technology acceptance models), allow the integration of these two motivational mechanisms in order to explain consequences of AI anthropomorphism. We will further on review these theoretical frameworks along the dimensions of effectance and sociality in order to develop an integrated framework for AI anthropomorphism.

### 1.2. Conceptual Research Design and Scope of Contribution

This article is a conceptual paper aimed at theory development by synthesizing literature on AI anthropomorphism in human–AI interactions and introducing an integrative conceptual model with propositions that guide future empirical research. Following established methodological guidance for conceptual research (Jaakkola, 2020; Hulland, 2020; Rocco et al., 2022), the paper adopts a phenomenon-driven research design with AI anthropomorphism as the focal phenomenon. The main objective is to develop a parsimonious mid-range theoretical framework that integrates previous research insights and explains how anthropomorphic perceptions of AI shape behavioral intentions through established antecedents of technology acceptance and threat appraisal. The paper draws on multiple domain theories that have been widely used to explain technology-related attitudes and behaviors, namely TAM (Davis, 1989), the TPB (Ajzen, 1991) and the TRM (Staw et al., 1981). These established technology acceptance models serve as the substantive knowledge base for describing how evaluative cognitions (e.g., perceived usefulness, behavioral control, subjective norms, ease of use, opportunities or threats) influence AI adoption or resistance. Although these frameworks specify important predictors of behavioral intentions in relation to AI, they offer limited guidance on how anthropomorphic perceptions of AI are structured and translate into specific behavioral intentions. To address this limitation, this paper builds on the stereotype content model (SCM, Cuddy et al., 2008) and uses the warmth–competence distinction in social perception as ground for integrating diverse anthropomorphic cues and perceptions of AI anthropomorphism. By using warmth and competence as higher-order organizing constructs, the paper synthesizes the findings from the AI anthropomorphism literature and maps them onto the predictions of the technology acceptance and threat rigidity models. The aim of the paper is not to engage in an empirical validation, but to provide a theoretical integration and argumentation drawing on previous empirical and theoretical research to derive testable theoretical propositions that specify the relation between warmth and competence attributes ascribed to AI and the acceptance or resistance outcomes as described by existing technology acceptance models. Accordingly, the key contribution of this paper is the development of a theoretically grounded integrative model that generates testable predictions and clarifies when anthropomorphic features ascribed to AI are likely to foster acceptance, trigger resistance or produce ambivalent responses. Empirical testing and validation of the theoretical propositions and their associated corollaries are explicitly positioned as avenues for future research.

### 1.3. Technology Acceptance Models

Although the TPB (Ajzen, 1991) and TAM (Davis, 1989) were studied as separate theoretical frameworks to predict attitudes and behavioral intentions in different domains, they share some common antecedents for the two key variables. It is our contention that when applied to AI, perceived behavioral control (PBC—TPB) and perceived ease of use (PEU—TAM) are related to effectance (Epley et al., 2007) and capture the same dimension, namely participants’ expectations that they can easily use AI in their daily lives. This claim is supported by the very high positive correlation (r = 0.64) reported between PEU and PBC (Cheng, 2019). Also related to AI effectance is perceived usefulness (PU-TAM), describing the expectation that using AI will ultimately benefit performance and improve general effectiveness (King & He, 2006). Finally, subjective norms (SN- TPB) are related to sociality (Epley et al., 2007) and refer to the shared social perception that using AI is beneficial and socially accepted. Previous meta-analytic evidence on TPB as well as TAM has supported the predictive value of these dimensions for attitudes and behavioral intentions (King & He, 2006; Weigel et al., 2014). We argue that attributes that capture sociality relate to the social nature of interacting with AI, while attributes that capture effectance relate better to its nature as a cognitive tool.

An alternative model that explains behavioral intentions related to technological advancements or more general changes is the TRM (Staw et al., 1981). In line with the TRM, the introduction of AI can, in principle, be perceived as bringing substantial opportunities (increased effectiveness, boosting performance, convenience, all reflective of effectance) as well as substantial threats (replacing jobs, taking over control of tasks, increasing dependence, all reflective of low sociality) (Belloni & Curșeu, 2025). Grounded in the stimulus–response perspective, the TRM predicts that perceived threats will trigger cognitive rigidity, limit the depth of information processing and elaboration in relation to AI and trigger defensive reactions, while perceived opportunities will increase flexibility of information processing (Staw et al., 1981) and trigger adoption and engagement with AI. Most of the research grounded in TRM has focused on examining how employees perceive artificial intelligence and the likelihood that its advancement will alter the work environment and potentially supplant their status within it (Belloni & Curșeu, 2025; Mirbabaie et al., 2022; Ursu et al., 2025). In cases where employees perceive their competencies as susceptible to substitution, they have higher sensitivity toward the threat posed by AI to their professional identity within the workplace (Belloni & Curșeu, 2025; Selenko et al., 2022). This susceptibility persists even in collaborative contexts, contributing to a palpable sense of identity diminishment among employees (Harris-Watson et al., 2023). Moreover, the emergence of generative AI presents challenges within academic spheres as well, being regarded as a mechanism that undermines and jeopardizes intellectual property rights (Eke, 2023). Furthermore, of particular concern is the capacity of software such as ChatGPT to generate extensive volumes of original, yet scientifically unverified texts, requiring the resolution of misinformation issues to mitigate the potential threat posed by AI to diverse societal facets (De Angelis et al., 2023). In line with these results, we argue that the key variables that predict intention to use (SN, PBC, PU, perceived opportunities and threats) can be derived from the anthropomorphic attributes ascribed to AI.

Some empirical studies have already explored the association between AI anthropomorphic features and TRM as well as TAM variables (Belloni & Curșeu, 2025). A stream of research that manipulated the physical appearance of the robots or chatbots as an indicator of anthropomorphism, typically using a manipulation check that referred to the perceived similarity with human appearance, revealed inconclusive effects with respect to PU or PEU (J. Kim & Im, 2023). In a systematic investigation across four experiments, S. Y. Kim et al. (2019) showed that human-like physical appearance of robots increased perceived warmth, yet had no significant association with perceived competence of robots. Moreover, meta-analytic evidence shows that anthropomorphic features ascribed to service robots have a positive and significant effect on perceived ease of use, usefulness and intention to use (Blut et al., 2021). Finally, empirical results presented in J. Kim and Im (2023) show that perceived cognitive intelligence is the strongest predictor of anthropomorphic response in relation to AI, followed by perceived emotional intelligence and human-like appearance. In the following section, we build on general social categorization and interpersonal perception processes to integrate research on anthropomorphic features typically ascribed to AI and to derive theoretical propositions concerning their relation with SN, PU, and PBC as well as perceived opportunities and threats.

## 2. An Integrative Model of Anthropomorphism in AI

In order to integrate the insights related to AI anthropomorphism, we build on two theoretical frameworks. First, according to the modality-agency-interactivity-navigability (MAIN) model (Sundar, 2008), interface cues play a significant role in shaping user perceptions by activating mental shortcuts and expectations, including stereotypes. In line with MAIN, we argue that readily detectable AI interface cues (robotic facial features, voice, name) trigger social categorization processes in line with the general tendencies identified in interpersonal interactions (Kinzler et al., 2010). Second, we build on the CSAT (Nass et al., 1995) to argue that interaction with any artificial tool triggers anthropomorphic expectations. In a study that compared chatbots with interactive websites, Ischen et al. (2020) show that participants equally ascribe anthropomorphic features when interacting with chatbots and websites. In line with this theoretical framework and results, we build on the interpersonal perception literature to argue that warmth and competence (Fiske et al., 2007) are the key dimensions that can capture anthropomorphic features users tend to ascribe to AI.

A first component in the evaluation of human–AI interaction refers to competence. With its higher power of processing big sets of data, resulting in superior expertise compared to any other agent, AI is often seen as superior to humans (Ramaul et al., 2026). Intelligence, notwithstanding the concerns it provokes, remains one of the best predictors for social presence, personalization and satisfaction (Verhagen et al., 2014), this occurring because competence increases consumers’ cognitive-based trust (Yen & Chiang, 2021). Additionally, self-disclosure and reduced privacy concerns can be encouraged by behaviors such as planning actions or anticipating problems, intellectual abilities that foster safety in the interaction (Gieselmann & Sassenberg, 2023). In competitive contexts, receptivity of an AI system as a new team member is related to numerous traits, but the relationship between competence and acceptance is particularly strong, encompassing adapting team processes to a newcomer, knowledge utilization and psychological acceptance (Harris-Watson et al., 2023). Given that competence is frequently examined through the lens of perceived utility, we believe it is predominantly related to the effectance motivation (Epley et al., 2007, 2008). In other words, anthropomorphism enhances self-efficacy by understanding AI as a cognitive tool and ultimately being able to jointly understand and control the environment through solving problems and making better decisions.

A second motivational mechanism concerns the relation between ascribed warmth and sociality motivation, as anthropomorphism can augment the perception of social connectedness with AI, consequently reinforcing its relevance in fulfilling affiliative needs (Epley et al., 2007). We argue that the second type of anthropomorphic feature ascribed to AI, focused on social engagement or warmth (the degree to which the conversation is led by friendliness, interactivity, authenticity) is predominantly related to sociality. Studies suggest the fact that physical characteristics, such as a human mouth, a large smile, or a feminine face, can lead to perceptions of warmth and friendliness, and increase levels of comfort for the user (Castro-González et al., 2018). Friendlier robots have a significant impact on the service value, increasing it depending on the customer’s need for social interaction (Belanche et al., 2021). Also, mediated by social presence, the caregiver quality of the AI leads users to form more positive attitudes toward technology, and to feel greater satisfaction toward the usefulness of robots (K. J. Kim et al., 2013).

Interestingly, Go and Sundar (2019) indicate that an AI system low on visual anthropomorphic cues can still be positively perceived if it is highly interactive in a chatting context. Nevertheless, this phenomenon does not manifest in situations where the chatbot is presented as a human, potentially attributable to heightened expectations arising from interactions with actual humans (Sundar et al., 2015). Furthermore, empathy (Pelau et al., 2021) and contingency (the extent to which the robot shows understanding of customer responses, Schuetzler et al., 2020), are two of the moderators in the relationship between perceived anthropomorphism and acceptance or trust in AI. Warmth and competence ascribed to AI guide the human–AI interaction, and the alignment between the human and AI goals plays a key role as a boundary condition in this association (McKee et al., 2023).

Social interdependence theory (SIT, Deutsch, 1949) distinguished three forms of goal interdependence: positive goal alignment leading to collaborative attitudes, negative goal alignment leading to competitive attitudes, and independent goals. AI tools naturally cluster along these three forms of social interdependence with assistive AI technologies, typically recognized as collaborative tools, algorithmic trading rivals and AI opponents in chess games, recognized as competitive agents, while autonomous AI systems (e.g., autonomous driving) are perceived as rather independent in terms of goals alignment. For the sake of simplicity, we will use AI autonomy as a criterion for positioning AI agents in the social interdependence framework and argue that assistive AI systems are typically perceived as collaborative agents, while autonomous AI systems are associated with perceptions of goal independence or, in the most extreme cases, competitive agents (Formosa, 2021).

Warmth ascribed to AI is particularly important in assistive contexts (e.g., customer service, tutoring) because communal cues align with users’ expectations of care, guidance and cooperation; therefore, we expect that communal attributes foster PBC. However, in contexts in which AI is granted full decision-making authority (e.g., autonomous driving, algorithmic hiring), communal attributes could be reinterpreted as displays of pseudo-empathy and thus reduce PBC. This distinction underscores an important boundary condition of the relation between communal attributes and PBC, such that communal attributes ascribed to autonomous rather than assistive AI actors may backfire against PBC. Competence attributes ascribed to AI enhance perceptions of PU as well as perceived opportunities in relation to using AI, as users tend to trust more competent rather than incompetent interaction partners. However, in line with recent findings on sociopathic features ascribed to AI (Belloni & Curșeu, 2025; Olar et al., 2026), under conditions of high autonomy, overly competent AI can also be perceived as domineering, potentially manipulative and ultimately status-threatening. Therefore, while competence cues generally strengthen PU and opportunity perceptions, over-competence could be perceived as cognitive superiority and also trigger threat perceptions when AI is an autonomous actor, rather than a controllable tool. Given these arguments, we formulate the following propositions:

**Proposition** **1.** *Interpersonal warmth attributes ascribed to AI (e.g., empathy, friendliness) increase PBC and reduce perceived threats in relation to AI, and the effect is stronger for assistive AI systems (e.g., customer service, education) than for autonomous AI contexts (e.g., autonomous decision-making), as under high autonomy conditions, warmth attributes could be interpreted as “pseudo-empathy”.*

**Proposition** **2.** *Competence attributes ascribed to AI (e.g., competence, problem solving, authority) increase PU and opportunities perceived in relation to AI, and the effect is stronger for assistive rather than autonomous AI contexts, as under high autonomy conditions, competence cues ascribed to AI can also increase threat perceptions triggered by perceived cognitive superiority and fear of domination and status replacement*.

**Figure 1:**
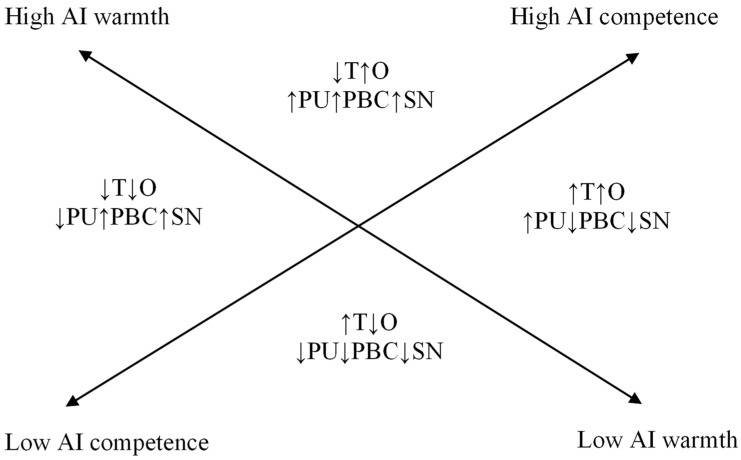
An integrative framework for the predictions derived from the warmth and competence dimensions in AI anthropomorphism. Legend: AI = artificial intelligence; T = threats; O = opportunities; PU = perceived usefulness; PBC = perceived behavioral control; SN = subjective norms; ↑ = high; ↓ = low.

Derived from the propositions stated above, we theorize significant interaction effects between the two dimensions of interpersonal perception (warmth and competence) and the perception of AI, interaction effects that are depicted in Figure 1 and summarized in the following corollaries:

**Corollary** **1.** *A combination of high warmth and high competence leads to high PU, PBC, SN and opportunities and respectively low threats in relation to AI.*

**Corollary** **2.** *A combination of low warmth and low competence leads to low PU, PBC, SN and opportunities as well as high threats in relation to AI.*

**Corollary** **3.** *A combination of high warmth and low competence leads to low PU, perceived threats and opportunities as well as high PBC and SN in relation to AI.*

**Corollary** **4.** *A combination of low warmth and high competence leads to increased PU, threats and opportunities as well as low PBC and SN in relation to AI.*

Figure 1 depicts an overall model of anthropomorphic features in AI in relation to the antecedents of intention to use, as described in TPB, TAM and TRM.

### Social Categorization Cues in AI Anthropomorphism

In human–robot interactions, subsidiary elements such as facial features, name, image, gender or human voice are essential in enhancing trust (Waytz et al., 2014), primarily by promoting a sense of social presence (J. Kim & Im, 2023). We build on social categorization research showing that gender, race and age are primary social categorization tendencies in humans, starting with early childhood (Kinzler et al., 2010), and discuss the attribution of such specific attributes to AI. Our key argument is that specific stereotypical representations are used to assess “the humanness of AI” based on social categorization cues that users derive from the use of voice or other human-like features that are embodied in AI tools (e.g., facial appearances of robots, language). In our framework, these categorization cues act as antecedents of warmth and competence perceptions, two universal dimensions of social categorization (Fiske et al., 2007) which then shape acceptance outcomes differently, depending on whether AI is assistive or autonomous.

With regard to gender, research revealed that female chatbots engaging in social behaviors elicit favorable consumer responses, even under error conditions (Watkins, 2021). Furthermore, female virtual assistants are markedly more prone to forgiveness for errors in comparison to their male counterparts (Toader et al., 2019). Stroessner and Benitez (2019) reported that feminine artificial robot faces generated assessments of greater amiability, thereby eliciting heightened inclinations toward interaction. Meta-analytic evidence on service robots shows that robots sharing female physical appearance elicit more anthropomorphic reactions and intention to use as compared to their male-looking counterparts (Blut et al., 2021). One possible rationale for this phenomenon could be rooted in the alignment between the designated role of virtual voice assistants and the nurturing, compassionate disposition traditionally associated with females, characterized by attentiveness to the needs of others. Experimental results support this explanation and show that general social categorization tendencies and inference of gender stereotypical attributes apply to robots (Eyssel & Hegel, 2012). Thus, gendered voices and appearances can be read as antecedents of warmth attributions, and their positive effect is strongest in assistive AI contexts such as tutoring or customer service. However, when the same warmth cues are displayed by autonomous AI systems with decision-making power, they may be reinterpreted as pseudo-empathy, illustrating the boundary condition of the type of AI–human interaction we outlined in Proposition 1. Perceived age similarity with an AI-powered educational instructor (as inferred from voice) fosters credibility and increases the perception of social presence and motivation to learn in students (Edwards et al., 2019). Such results suggest that similar social identification mechanisms that explain interpersonal interactions in humans are also applicable to explain interactions with AI-powered systems.

In line with such claims, research regarding the age of voice assistants suggests that young users prefer exclusively youthful AI interfaces. Conversely, middle-aged and older adults can accommodate either youthful or middle-aged artificial assistants, albeit not surpassing their own age bracket in preference (Zhong et al., 2024). Trust as a function of voice assistant age has been explored as well, with investigations revealing that this dimension is surpassing the characteristic of gender in terms of impact on perceptions and attitudes (Watkins, 2021). The intriguing phenomenon of favoring young adults, particularly in intelligent home systems, could potentially stem from the perception associated with this demographic, incorporating attributes such as agility, information proficiency, altruism, optimism, and engendering a sense of connection with external events (Watkins, 2021). In terms of social categorization, youthful cues tend to be mapped onto competence (speed, proficiency), whereas older voices invite warmth (wisdom, care). Hence, age-based categorization captures how competence and warmth are not abstract, but socially coded, supporting our argument in Proposition 2 that competence cues generally enhance usefulness and opportunity perceptions, while at extreme levels also raising status-related threat concerns in highly autonomous contexts.

Furthermore, studies have identified a preference for intra-racial interactions, as evidenced by the superior performance of white participants in engagements with racially congruent white AI counterparts, while Black participants demonstrated heightened efficacy when engaging with AI systems characterized as “black” (Atkins et al., 2021). It is imperative to acknowledge the corpus of research investigating stereotypes and discriminatory tendencies within AI systems that lends support to the supposition that appearances diverging from Caucasian norms tend to provoke more overt instances of dehumanizing commentary. Notably, AI representations featuring Black or Asian traits were subjected to approximately twice the frequency of objectifying and stereotypical remarks compared to AI racialized as white (Strait et al., 2018). Although further investigation is warranted, this inclination to attribute intelligence and personhood based on racial markers may derive from the dangerously inaccurate presumption that whiteness constitutes the default standard of human identity. This bias within the technology industry can be mitigated through the promotion of diversity concerning racial representation and the implementation of comprehensive training for AI systems to equitably acknowledge and address various ethnic groups with equivalent precision. In our model, racial similarity acts as a cue of warmth and trust that increase PBC and reduce threats, especially in assistive interactions. Yet when racial similarity is paired with high warmth and competence in autonomous AI, it may shift from bonding to identity threat through unfavorable social comparisons.

With respect to language, the findings in this direction can be divided into benefits of verbal and non-verbal cues adopted by the robot. Firstly, embracing a literal linguistic style would enhance the perceived credibility of the system, targeting the cognitive facet of trust (Choi et al., 2019), whereas an informal style heightens both mindless and mindful perceptions of its anthropomorphic attributes. As AI increasingly adopts human-like characteristics, and engages in behaviors such as self-disclosure, the user’s responses to sensitive questions tend to align more closely with socially desirable norms, and thus less honest, mirroring a phenomenon observable in human-to-human interactions (Schuetzler et al., 2018). Nonetheless, greeting, personal interaction and politeness through words are antecedents of humans’ relational decisions, in terms of the time spent in the interaction, and the likeliness to reopen a conversation in the future (Cafaro et al., 2016). These linguistic choices directly prime either warmth (through friendliness, enthusiasm, politeness) or competence (through formality and credibility). Such duality reinforces our propositions that warmth enhances acceptance in assistive contexts but may appear insincere in autonomous AI, whereas competence through language enhances usefulness but can also heighten threat perceptions when autonomy is high.

In relation to the non-verbal cues, an interaction with a high-pitched, enthusiastic, female voice was found to be significantly more appealing and entertaining than a conversation with a lower, stronger toned voice (Niculescu et al., 2013). In AI assistive contexts, interpersonal similarity signals goal alignment and cooperation (positive interdependence); therefore, it is likely to enhance PBC and reduce perceived threat. However, in autonomous AI contexts, interpersonal similarity may trigger identity threat, as users may perceive AI as a threat to their status and expertise and as a competitor rather than collaboration partner (negative interdependence). In autonomous AI contexts and when AI is perceived as overly warm and competent, interpersonal similarity no longer assures but threatens and amplifies unfavorable social comparison, potentially leading to resistance rather than acceptance. In line with these arguments, we formulate the following proposition:

**Proposition** **3.** *Perceived similarity between the user and AI with respect to perceived age and race increases PBC and reduces perceived threats in relation to AI, and the effect of similarity is stronger for assistive rather than autonomous AI contexts, as when AI is perceived as overly competent and warm, similarity can foster identity threat through unfavorable social comparisons, leading to rejecting rather than accepting AI.*

## 3. Future Research Directions and Practical Implications

The conceptual framework introduced in our paper aims to tackle some of the common concerns related to the heterogeneity of definitions, operationalizations and consequences of AI anthropomorphism identified in recent reviews (Chaturvedi et al., 2025; M. Li & Suh, 2022; T. Li et al., 2025; Ramaul et al., 2026). The integrative framework builds on prior models of technology acceptance, while concurrently considering age, gender, race, and language as social categorization cues used in human–AI interactions. In line with the best practices for conceptual papers (Hulland, 2020; Jaakkola, 2020; Rocco et al., 2022), the proposed framework synthesizes insights from previous studies, reconciles mixed findings regarding the effects of AI anthropomorphism and delineates boundary conditions related to AI autonomy and social interdependence in human-AI interactions. By doing so, the paper not only integrates prior research but also provides a structured agenda for future empirical work aimed at testing the propositions across various contexts, task domains and levels of AI agency.

We theorize anthropomorphism as an specific mechanism in human-AI interaction that simultaneously enables adoption by activating perceptions of interpersonal opportunities and threats. Unlike other digital systems, AI is attributed with agency, intentionality and moral reasoning, leading users to evaluate it as a social autonomous actor rather than merely a tool. By building on the tenets of TRM, we highlighted how anthropomorphic cues trigger dual pathways: enhancing trust and usefulness (through opportunity perceptions), or amplifying concerns of manipulation and autonomy loss (through threat perceptions). Furthermore, we introduced perceived similarity that triggers social identity cues between user and AI as a novel mechanism explaining AI acceptance. Conceptual work in organization and management research (Ramaul et al., 2026) emphasizes the need to avoid reifying AI as a human actor and to avoid reductionist anthropomorphic language assumptions, especially in AI development. Consistent with this call, our integrative model treats warmth, competence, agency and moral agency as perceived features ascribed by users to AI and not as inherent technological properties of AI systems. Previous reviews in the literature (M. Li & Suh, 2022; T. Li et al., 2025; Chaturvedi et al., 2025) also acknowledge that some of the negative anthropomorphic AI features may backfire in certain contexts, yet they catalogue plausible moderators rather than offering an overarching theoretical framework to explain such boundary conditions. Our integrative framework contributes a mid-range theory of AI anthropomorphism with sharper boundary conditions, as it applies to socially interactive AI systems (chatbots, voice assistants, embodied robots), rather than computational back-end systems, as the theoretical propositions may differ across assistive and agentic AI systems. These theoretical propositions generate testable predictions about when and why anthropomorphism promotes AI acceptance versus resistance tendencies in users, thereby offering a more generative account than the previous technology acceptance models in isolation.

In terms of theoretical implications, our model integrates the insights of the TPB and TAM as key theoretical models that explain behavioral intentions in relation to technology, with effectance and sociality motivational mechanisms that explain AI anthropomorphism (Epley et al., 2007, 2008). Moreover, we integrate these two dimensions with the threat rigidity framework that distinguishes between opportunities and threats in relation to technology to explain more broadly how anthropomorphism impacts attitudinal and evaluative tendencies in relation to AI. We argue that ascribing human-like attributes to AI is a key precursor of perceived usefulness, subjective norms, attitudes and perceived behavioral control in relation to AI. Humanizing AI means that interpersonal perception mechanisms explain intention to use and ultimately the adoption of AI. The boundary conditions formulated in the first two propositions account for why anthropomorphic AI cues sometimes backfire when warmth is interpreted as pseudo-empathy and competence as cognitive superiority. Moreover, we argue that interpersonal similarity may trigger identity threat when AI is autonomous and perceived as overly warm and competent. Future research could build on these propositions in order to examine non-linear effects (e.g., very high warmth leading to distrust, very high competence leading to threat perceptions), as well as the moderating role of social categorization cues such as gender or race in different cultural contexts. These boundary conditions are unique to AI because it is attributed with agency, intentionality and moral reasoning, unlike other systems that are merely controllable tools.

A first line of research could explore specific warmth and competence attributes that are typically used to describe interactions with AI across different areas of application. It could be that not all warmth and competence attributes are equally used in the case of AI, and future research could shed more light on such differences. We could expect that AI used as support or recommendation tool will be ascribed communal rather than agentic attributes, while AI used as a decision-making tool will be ascribed agentic rather than communal attributes. Experimental manipulations of warmth and competence cues across assistive and autonomous AI systems would therefore provide critical empirical validation for our propositions. Another line of research could explore interpersonal similarity as a driver of AI acceptance and intention to use. We expect that perceived interpersonal similarity with AI agents fosters social bonds and increases the quality of social connection. Building on perceived interpersonal similarity, a relevant avenue for future research could be the exploration of the social identity tenets in human–AI interactions. As previous research shows that participants tend to identify equally with human and AI communication partners (Mirbabaie et al., 2021), a key question arises: namely, do we use similar principles for social categorization in relation to AI agents as we do with humans, or are such processes different? In a study that manipulated identification with AI, Cao et al. (2023) showed that participants in the experimental condition experienced more psychological entitlement; therefore, some support for the esteem-boosting role of AI already exists. The subsequent question is how does the minimal group perspective impact the perceived attractiveness of social robots or other AI-powered robots? Preliminary research shows that similarly to human experts, the AI-recommending systems are trusted especially when they are perceived to be in- rather than out-group (J. Wang et al., 2020). Moreover, when AI is perceived as an “outgroup” that threatens professional identity, status and job security, similar social distancing patterns are engaged, as extensively illustrated in the case of identity threat in intergroup relations research (Mirbabaie et al., 2022). Our propositions related to the perceived threats and opportunities capture such social identification effects, and future research could investigate the extent to which categorizing AI-powered systems as in-group versus out-group members impacts perceived opportunities and threats related to interactions and use. To conclude, in order to achieve emotional connection with the brand and satisfaction with the product (Araujo, 2018), companies could focus on interface aspects of anthropomorphism in order to capitalize on beneficial consequences of social categorization processes that are automatically triggered in users, yet companies should not disregard the negative consequences of social categorization in terms of reinforcing and maintaining discriminative tendencies in users. In practice, this means that designers should carefully tailor anthropomorphic cues to the level of autonomy (warmth in assistive contexts, competence in autonomous contexts) and ensure diversity in representation to avoid reinforcing negative social stereotypes. Policymakers can also encourage guidelines for responsible anthropomorphism, requiring organizations to test for unintended effects such as over-trust, pseudo-empathy, or identity threat before deployment.

A second line of research pertains to the propositions related to the warmth and competence dimensions of AI perception. One of the key objectives of our paper was to consolidate research efforts within this domain and provide the AI industry with a comprehensive tool for better comprehension of the attributes fostering user acceptance and trust. We argued that warmth and competence are key dimensions used to describe AI and future research could identify the key attributes that are used in these two dimensions. Parsimonious scales were extensively used in the literature to assess warmth and competence (Fiske et al., 2007), yet the tendency of these short instruments was to only use positive attributes (Cuddy et al., 2008), assuming that a low score would be indicative of the opposite pole of the evaluating dimension. We propose that using a semantic differential approach would generate more fine-grained evaluations for competence and warmth and allow the use of bipolar attributes that would capture both poles of the two core evaluative tendencies. Based on the attributes used in previous research (Cuddy et al., 2008; Ho & MacDorman, 2010), such a semantic differential for evaluating AI features could include, for warmth, pairs such as unfriendly—friendly, malevolent—well-intentioned/empathic, hostile—trustworthy, immoral—righteous, dominant—tolerant, useless—helpful, or dishonest—sincere, while for the competence dimension, incompetent—competent, unintelligent—intelligent, ignorant—knowledgeable, unimaginative—creative, foolish—wise.

Hypotheses derived from the uncanny valley theory (S. Wang et al., 2015) or technology threat avoidance theory (Liang & Xue, 2009) suggest that customers’ perception and trust are fragile, and diminished by the excessive infusion of accentuated human characteristics like intelligence or expertise, without compensation of empathy (Pelau et al., 2021). In addition, research also emphasizes that users also tend to ascribe sociopathic features to AI (Duffy, 2003; Liang & Xue, 2009; Belloni & Curșeu, 2025; Olar et al., 2026), and future research could further explore how such tendencies relate to the perceived warmth and competence as described in our general model. In line with the corollaries of our model, future research could test non-monotonic relations between warmth and competence on the one hand and intention to use AI on the other hand. Extreme levels of competence and warmth could reduce intention to use when they are related to low perceived opportunities or high AI threats that could indicate AI manipulative intent. Another prediction that could emerge from our model is that AI may be perceived as cold or lacking empathy (sociopathic features, Belloni & Curșeu, 2025; Olar et al., 2026), thus deeply threatening when competence is extremely high and warmth is extremely low. Given that sociopathic features may encapsulate a very specific combination of low warmth and high competence, we expect that sociopathic attributes ascribed to AI have additional predictive value for AI-related attitudes and behavioral intentions, after controlling for warmth and competence. Our model also integrates recent empirical findings showing that perceived threats and opportunities in relation to AI jointly impact performance enhancement expectations (Belloni & Curșeu, 2025) and reliance on AI-generated advice (Olar et al., 2026). We thus build on the TRM to argue that perceived warmth and competence are the underlying dimensions that trigger threat and opportunity perceptions in relation to AI and call for more empirical research that investigates the interplay of these two TRM dimensions as predictors of behavioral intentions in relation to AI. In addition, our model can be further extended to include additional effects, such as resistance to anthropomorphism stemming from humans’ innate desire for autonomy and self-guided problem-solving strategies, or the effect of users’ internal states, encompassing their experiences and expectations, factors that introduce complexity and variability in the predictions of the aforementioned model.

## 4. Conclusions

The paper develops a mid-range theoretical model that integrates the empirical findings on anthropomorphism in relation to AI, using an orthogonal framework of competence and warmth. We combine insights from established technology acceptance models to formulate three propositions and four corollaries on the consequences of ascribing specific human attributes to AI. Enhancing comprehension of both the challenges and opportunities presented by AI is essential for the formulation of effective design principles to increase acceptance and comprehensive policies for using AI. Given the enduring presence of AI, it is important to cultivate the knowledge necessary to coexist harmoniously with this technology and to foster synergies and resilience in human–AI ecosystems. 

## Data Availability

No new data were created or analyzed in this study. Data sharing is not applicable to this article.

## Abbreviations

The following abbreviations are used in this manuscript:

AI	Artificial Intelligence
CSAT	Computers as Social Actors Theory
TPB	Theory of Planned Behavior
TAM	Technology Acceptance Model
TRM	Threat Rigidity Model
SCM	Stereotype Content Model
PBC	Perceived Behavioral Control
PEU	Perceived Ease of Use
PU	Perceived Usefulness
SN	Subjective Norms
SIT	Social Interdependence Theory
MAIN	Modality-Agency-Interactivity-Navigability
